# Lunasin-like Peptide in Legume and Cereal Seeds: A Review

**DOI:** 10.3390/ijerph22101505

**Published:** 2025-09-30

**Authors:** Jorge Oswaldo Gutiérrez-López, Erick Damián Castañeda-Reyes, Gloria Dávila-Ortiz

**Affiliations:** Protein of Vegetal Origin Laboratory, Biochemistry Engineering Department, Escuela Nacional de Ciencias Biológicas, Instituto Politécnico Nacional, Av. Wilfrido Massieu s/n, Zacantenco, Gustavo A. Madero, Mexico City 07738, Mexico; jgutierrezl0701@egresado.ipn.mx (J.O.G.-L.); edecare@gmail.com (E.D.C.-R.)

**Keywords:** lunasin-like peptide, lunasin, bioactivity, legume seeds, cereal seeds, anticancer, antioxidant, antibody

## Abstract

Lunasin is a peptide found in the soybean albumin 2S subunit, which has important bioactivities, such as anticancer and antioxidant. Recently, peptides similar to soybean lunasin have been reported in other cereal and legume seeds; for this reason, it is considered important to carry out a review that compiles this information, whose interest lies mainly in the bioactive properties of these peptides. The peptides reported in the literature contained in barley, wheat, rye, triticale, oat, black nightshade, amaranth, bean, chickpea, grass pea, lentil, and pea are analyzed and described. Isolation methods such as ion exchange chromatography, immunoaffinity column chromatography, Western blot, reversed-phase chromatography coupled to an electrospray ionization source, extraction with water and dialysis, and extraction with PBS, and tests such as internalization, radical scavenging, chelating, cytotoxicity in cancer cell lines essays, and histone acetyltransferase inhibition essays were carried out to identify their anticancer properties. It is worth mentioning that the in silico analyses of proteins in which the lunasin-like peptide is located have been developed in some of these seeds; however, more studies are needed in order to confirm sequence similarity to that of the lunasin peptide. Further work is needed in order to identify the sequence of these lunasin-like peptides and corroborate their similarity to that of the lunasin, such as the development of specific antibodies for each lunasin-like peptide reported in each type of seeds. This document aims to compile the advances in the research on lunasin-like peptides and their bioactivities to have a better understanding of the current advances related to these peptides.

## 1. Introduction

Research on plant-based active compounds has increased in recent years due to their benefits for human health, and there are currently compounds that come from plants or synthetic derivatives of plant compounds, such as peptides, polyphenols, and terpenoids, that are used as drugs [1,2]. These types of compounds have advantages over current analogous drugs, presenting fewer or no side effects for health and providing a possible alternative for their use in preventing diseases and even as treatments; in addition, they can be used in the common diet or integrated into the production of functional foods, in which they can exert their bioactivity [1].

Seeds have been heavily studied due to the presence of various bioactive compounds with the potential to be used for the benefit of human health. For example, different peptides from legumes and cereal seeds have been reported to show antidiabetic, antioxidant, antiobesity, anticancer, antimicrobial, antihypertensive, and anti-inflammatory bioactivities [2,3,4,5,6]. Methods used for the extraction of proteins from seeds include alkaline extraction, ultrasound-assisted extraction, extraction by the fractionation method, enzymatic extraction, or enzyme-assisted microfluidization [1,2].

An example of a biological bioactivity peptide is lunasin, which has been attributed with anticancer, anti-inflammatory, and antimutagenic activities in in vitro and in vivo experiments [7,8,9]. The lunasin properties have been associated with the sequence and structure; its carboxyl terminus contains nine Asp (D) residues, an Arg-Gly-Asp (RGD) cell-binding motif, and a helix-loop-helix with structural homology to chromatin-binding proteins. Some authors have reported the presence of peptides similar to lunasin (lunasin-like) in barley [10], wheat [11], amaranth [12], and more recently in maize [13]. In recent years, new molecules with no or a small chance of side effects have been studied for different diseases, including cancer.

Cancer is a disease that affects millions of people worldwide. Moreover, several of the available treatments often have side effects in patients, which raises the problem of obtaining treatments with lower risks of presenting such effects; therefore, new alternatives continue to be sought [14]. Lunasin and lunasin-like peptides could be used in the treatment or prevention of this chronic disease because of their bioactive activities previously mentioned [12,15,16].

The objective was to compile the advances in the research on lunasin-like peptides and their anticancer, anti-inflammatory, and antimutagenic activities in order to have a better understanding of the current state of the art related to this peptide.

## 2. Lunasin in Soybean

**Figure 1:**
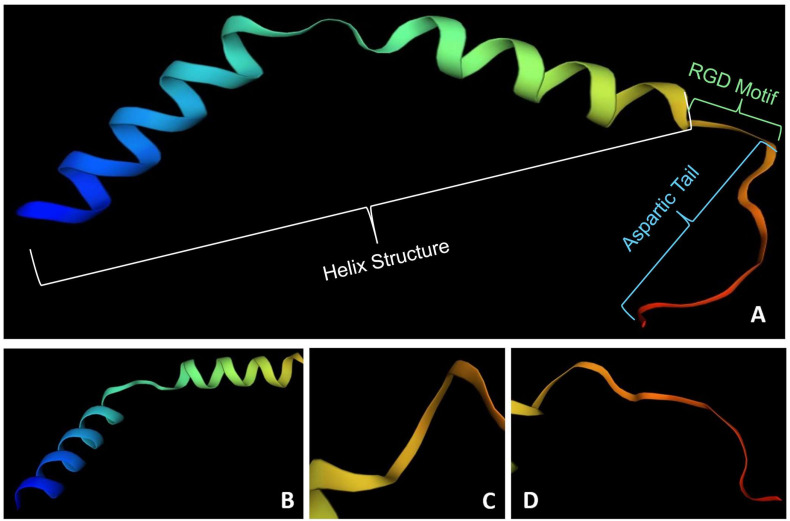
Lunasin 3D structure. (**A**): Lunasin complete 3D structure; (**B**): N-terminal 3D structure of the lunasin peptide; (**C**): RGD cell-binding motif; (**D**): C-terminal 3D structure of the lunasin peptide. This model was conducted in silico using the sequence from Davies et al. and using the PyMOL software [36,37].

Lunasin is a soybean-derived peptide [17,18] that has been shown to have anticancer effects [19]. This peptide exerts anticancer effects through multiple mechanisms: it binds deacetylated histones H3/H4 and inhibits acetylation, thereby suppressing cell transformation [20]; it also reduces proliferation, induces apoptosis, enforces cell cycle arrest, and diminishes metastatic behavior in various cancer cell lines [21]. In vivo, rodent studies report reduced tumor incidence, delayed onset, and smaller tumor burden in skin, mammary, and colon carcinogenesis models [21,22,23,24]. Lunasin has additionally been shown to synergize with chemotherapeutics such as oxaliplatin and to attenuate pro-inflammatory and oxidative signaling, contributing to a less tumor-promoting microenvironment [25]. The strongest evidence comes from reproducible in vitro mechanistic studies, with supportive preclinical animal data, whereas clinical efficacy in humans remains unproven, as no trials have tested its anticancer activity, highlighting a clear translational gap. It contains 43 amino acid residues (Figure 1A), and it is characterized by 19 highly charged amino acids, including a sequence of nine consecutive aspartic acid residues at the C-terminal region (accession sequence AAB71140). The characterization of the cDNA encoding lunasin indicated that it corresponds to the 2S small subunit of soybean albumin [26,27]. 2S albumins are seed storage proteins that supply nutrients during germination and support seed defense. Conserved cysteine residues stabilize the proteins against temperature, pH, and proteolysis, while their α-helix-rich, positively charged structure underlies the antimicrobial activities reported for many members of this group [28].

The bioactive properties of lunasin are deeply related to its structure [29,30,31]. The structural data of lunasin allows for the evaluation of the biological activities that it presents and its interaction with other molecules within the human body, as well as the risks that could occur when consuming it [32]. Lunasin comprises several regions: the amino-terminal region, which is formed by 22 residues (Figure 1B), the central portion, which is similar to chromodomains of chromatin-binding proteins, the RGD motif, which allows for the internalization of the peptide (Figure 1C), and the aspartic acid tail in the carboxyl-terminal region (Figure 1D) [19,20,21,22,23,24,25,26,27,28,29,30,31,32,33]. Studies have shown different biological activities, such as anticancer [34], anti-inflammatory [35], and antimutagenic [20] correlated to the complete sequence of this peptide.

The RDG cell adhesion motif recognizes integrins related to progression, metastasis, and other cancer processes. In addition, it allows for the internalization of lunasin into cells and exerts antimutagenic activity [20,26,35]. On the other hand, the aspartic acid-rich tail has been related to anticancer activity, where studies have shown that this sequence of aspartic acid residues has the ability to inhibit histone acetylation through electrostatic interactions between the aspartic acid tail and H3 and H4 histones [26].

Some studies have linked the N-terminal region to the known effects of lunasin [20,34,35,38,39,40]. The sequence in the central portion of lunasin is similar to that of chromatin-binding proteins, and it is related to the chromatin-binding ability of lunasin and its antimutagenic effect [20,40]. This region increases the binding affinity for the inhibition of histone H4 acetylation [20]. It is also important for the antimutagenic effect, and exhibits some cytotoxicity against cancer cells [34,40]. In general, both the N-terminal and central regions are considered responsible for the antioxidant and immunomodulatory activities of lunasin [35,38,39]. Moreover, lunasin’s cancer-preventive properties have been demonstrated in a mammalian cell culture model and a mouse model of skin cancer against chemical carcinogens, oncogenes, and tumor suppressor protein inactivators [41].

Since lunasin was discovered in soybean, studies have focused on purifying and quantifying it from different soybean varieties, reporting concentration values ranging from 1100 to 14,000 µg lunasin/g of extracted protein [42,43,44,45,46]. The results obtained in these studies demonstrate that soybean genotype is the main factor affecting the amount of lunasin in soybean seeds, indicating the possibility of selecting and improving soybean varieties with a higher content of this peptide [42]. Environmental factors, mainly temperature and soil moisture, have also been found to affect the concentration of lunasin in soybean seeds. At the same time, light and dark conditions do not seem to have any effect [47]. Other factors, such as seed development and maturation stages, also significantly influence the lunasin content in soybeans. Seed maturation resulted in a significant increase in this content, while germination leads to a continuous decrease in lunasin with soaking time [43]. Some studies have concluded that germination time and temperature have significantly affected the composition and concentration of nutrients and bioactive compounds such as lunasin in germinated soybean meal from several Brazilian soybean cultivars [48,49,50]. To date, only differences in lunasin concentration across soybean varieties have been documented [45].

However, evidence indicates that lunasin can survive partial gastrointestinal digestion and reach systemic circulation, although absorption in humans is low and the intactness of the peptide remains uncertain. In healthy adults consuming 50 g/day soy protein, plasma levels peaked at ~50–110 ng/mL (~2–8% absorption) 30–60 min post-ingestion [20], while a triple-blind crossover trial in older adults taking 50 mg/day soybean extract detected low fasting levels (0–10 ng/mL) with no side effects [22]. Animal studies suggest higher absorption (~30%) when lunasin was administered with soy preparations [19], and in vitro Caco-2 models show that full-length lunasin and certain resistant fragments can cross intestinal monolayers, primarily via paracellular routes, with protease inhibitors like Bowman–Birk or Kunitz enhancing stability [32,51]. Key uncertainties include the fraction of lunasin absorbed in vivo, whether it remains intact or is fragmented, the influence of the food matrix, and the precise transport mechanisms. Overall, current data support partial gastrointestinal resistance and systemic exposure of lunasin or bioactive fragments, but the extent of biologically active peptides reaching target tissues remains unresolved.

## 3. Lunasin-like Peptide from Other Sources

**Figure 2:**
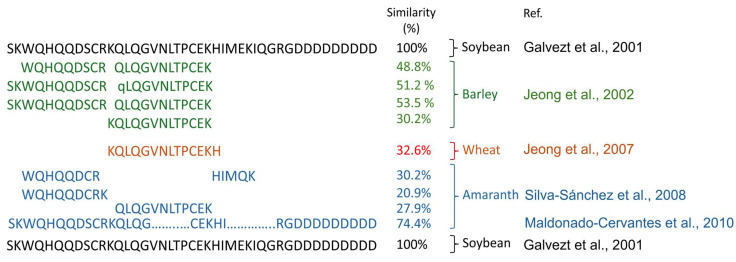
Similarity in the lunasin-like amino acid sequence of barley in green [10], wheat in red [11], and amaranth in blue [12,15] in comparison to that of soybean lunasin in black [20].

Once the biological activity of soybean lunasin was identified, studies were carried out to search for this peptide in various plants, which detected the presence of this peptide in variable amounts in different plants. This peptide has been reported in barley, wheat, rye, triticale, oats, black nightshade, amaranth, bean, chickpea, grass pea, lentils, and pea (Table 1); however, the primary structure of the native precursor protein differs from that of soybean lunasin, with some homology in their amino acid sequences (Figure 2). Several of these studies based the similarity of lunasin-like peptides on a positive anti-soybean-lunasin antibody test, instead of sequencing studies regarding the amino acid sequence of the lunasin; it is important to mention that the antibody recognizes part of the structure of the lunasin peptide, leading to concerns of proper identification. Therefore, there is a chance of a false positive, as it may be similar amino acid sequences that could be recognized by the anti-soybean lunasin antibody. Other studies based the similarity with lunasin on analyzing the molecular weight of both lunasin and lunasin-like peptides [10,11,12,52,53,54,55]. Nevertheless, it is suggested that additional experiments should be carried out in order to properly identify lunasin-like peptides, such as MS sequencing, to compare the sequence and structure of the peptides.

**Table 1:** Reports of lunasin-like peptide from diverse plant sources.

Source and Reference	Type of Bioactivity Study	Identification Techniques	Purification	Amino Acid Sequence	Activity or Analytical Test Results
Barley [10]	Studies in vitro in cell lines.	Western blot Matrix-Assisted Laser Desorption Ionization (MALDI)	Ion-exchange column chromatography	QLQGVNLTPCEK WQHQQDSC*R qLQGVNLTPC*EK QLQGVNLTPC*EK SKWQHQQDSCR SKWQHQQDSC*R KQLQGVNLTPC*EK q = Pyroglutamic acid C* = Amidomethyl-cysteine	Barley lunasin suppressed colony formation in ras (oncogene)-transfected mouse fibroblast cells induced with IPTG. They also inhibited histone acetylation in NIH-3T3 mouse fibroblasts and MCF-7 human breast cells in the presence of the histone deacetylase inhibitor sodium butyrate.
Wheat [11]	Studies in vitro with enzymes.	Western blot Liquid chromatography–electrospray ionization with mass spectrometry (LC-ESI-MS)	HPLC C18	KQLQGVNLTPCEKH	Lunasin obtained from wheat seeds dose-dependently inhibited histone acetylation (H3 and H4).
Rye [54]	Studies in vitro for digestion and in vivo in rats.	Western blot	HPLC C18	No data	Rye lunasin inhibited the activity of histone acetyltransferase (HAT).
Triticale	No data	Liquid Chromatography with Tandem Mass Spectrometry (LC-MS/MS)	HPLC	No data	Identification of the presence of lunasin peptide in triticale, wheat, and rye.
Wheat					
Rye [53]					
Oats [52]	Studies in vitro in cells and with enzymes.	Liquid Chromatography with Tandem Mass Spectrometry (LC-MS/MS)	HPLC	No data	Lunasin present in oats was found to have an antioxidant effect and a cell proliferation test in HEK 293 showed decrement of 20% in proliferation.
Black Belladonna [56]	Studies in vitro with enzymes and for digestion.	Western blot	Ion-exchange column chromatography	No data	Lunasin, isolated from SNL, inhibited core histone H3 and H4 acetylation, HAT activities, and Rb protein phosphorylation.
Black Belladonna [57]	Studies in vitro for scavenging effects.	Western blot	HPLC C18	No data	Lunasin from SNL protects DNA by chelating Fe^2+^, reducing its oxidation.
Amaranth [12]	Studies in vitro in cell lines.	Western blot Matrix-assisted laser Desorption Ionization coupled to an ion detector (MALDI-TOF) LC-MS/MS peptide de novo identification	Immunoprecipitation	HIMQK WQHQQDCR WQHQQDCRK QLQGVNLTPCEK	Trypsin-digested glutelin extracts showed induction of apoptosis against HeLa cells. Predicted other bioactive peptides in amaranth globulins and glutelins were mainly antihypertensive.
Amaranth [58]	Studies in vitro in cell lines.	SDS-PAGE	No data	No data	Amaranthus protein isolate was tested for its antiproliferative and antimutagenic effects in four cell lines (MC3T3E1 osteoblastic mouse calvaria-derived cells, UMR106 rat osteosarcoma-derived cells, and the Caco-2 and TC7 human colon-tumor lines), confirming these effects.
Amaranth [15]	Studies in vitro in cell lines.	Western blot PCR	Elution method	MTKFTILLISLLFCIAHTCSASKWQH-QQDSCRKQLQGFKMTATPPCEKHIT-RAFRRAPIQQRGISTRRGDDDDDDD-DDNHILSTRRDDEERTMRGRINYIRR-NEGKDPTPTLILREDE	The lunasin-like peptide is reported to internalize into the nucleus of NIH-3T3 cells in less time than soybean lunasin, inhibiting histone acetylation and the transformation of these cells into cancerous foci.
Amaranth [59]	Studies in vitro in cell lines.	SDS-PAGE MALDI-TOF		CAPYYLERWYRRKLF, EGDAZPGE, and GTFNE for unprocessed RPWWWHPGGGGGGGGLGAGT, HGSEPFGPR, RPRYPWRYT, and RDGPFPWPWYSH for extruded	Amaranth protein hydrolysates were tested in human and mouse macrophages that inhibited lipopolysaccharide-induced inflammation through inhibition of the NF-κB signaling pathway in THP-1 and RAW 264.7 cells; furthermore, extrusion enhanced this effect.
Amaranth [60]	Studies in vitro in cell lines.	SDS-PAGE	No data	No data	The anticancer effect of three amaranth protein hydrolysates was studied, proving their antioxidant and antiproliferative activity in MCF-7, A549, and HEK 293 cell lines. A549 and HEK 293 cell lines, with the tryptic hydrolyzate, were the ones with the highest anticancer activity.
Amaranth [7]	Studies in vitro in cell lines.	Western blot Tandem mass spectrometry	Elution method	No data	Changes in the proteomic profile of NIH-3T3 cells were analyzed in the presence of 3-methylcholanthrene, which was inhibited in the presence of lunasin-like. In addition, an NF-κB factor-related pathway of action produced by the amaranth lunasin-like peptide was revealed.
Beans Chickpea Grass pea Lentils Pea [55]	Studies in vitro in cell lines and in silico for sequences.	Western blot Immunoidentification HPLC coupled to NanoESI-MS/MS analysis Nano-LC–ESI–MS–MS	HPLC		Absence of lunasin before fermentation is reported; however, different bands of immunoreactive polypeptides were found. The number and intensity of lunasin-like polypeptides increased during sourdough fermentation. An inhibitory effect on the proliferation of human adenocarcinoma Caco-2 cells by sourdough extracts is reported.

### 3.1. Lunasin-like Peptide in Barley

Barley is a grass belonging to the *Poaceae* family and is mainly used as animal feed and in the production of alcoholic beverages, such as beer. In recent years, interest in barley has grown due to its various health effects, such as blood cholesterol reduction, glycemic index regulation, and antioxidant activity [61].

Lunasin was reported in barley. The peptide was identified by Western blot and mass spectrometry of the peptide in gel tryptic digest [10]. Then, lunasin was partially purified using anion-exchange and immunoaffinity chromatography. Subsequently, biological assays were performed with crude and purified lunasin (from 1 nM to 10 µM) in ras stably transfected mouse fibroblasts induced with isopropyl β-D-1-thiogalactopyranoside (IPTG), in which inhibition of colony formation was reported. In addition, these same fractions exhibited inhibition of histone acetylation in NIH-3T3 mouse fibroblasts and MCF-7 human breast cells in the presence of sodium butyrate, a histone deacetylase inhibitor [10]. In addition to that, a patent by the Andong University Industrial Cooperation Center in 2006 describes a method for isolating and purifying lunasin from soybean and barley with anticancer ability in skin cancer, applying it as a light-blocking agent together with other ingredients [62].

Jeong et al. [63] studied the bioavailability and bioactivity of barley lunasin present in different cultivars tested. The results showed that feeding rats with lunasin-enriched barley inhibited the activities of histone acetyltransferase (HAT), general non-depressible control 5 (GCN5), and P300/CBP-associated factor (PCAF) in the kidney and liver, confirming its bioactivity even after digestion. Furthermore, this purified barley lunasin localizes in the nucleus of NIH-3T3 cells, activating the expression of tumor suppressors p21 and p15 in the presence of monochloroacetic acid (MCA) [10]. As can be seen, the studies presented for barley lunasin are limited because they do not study the sequence of the peptide or the protein; only the masses are compared, and it is assumed that it is a lunasin-like peptide. In addition to that, Jeong et al. [10] proved different methods of purification for lunasin-like (ion-exchange chromatography with or without immunoaffinity chromatography); these methods proved to purify the lunasin peptide, presenting the same activity with no statistical difference. The difference between the patent methodology and these is that the patent proposes the use of three methods to purify the lunasin-like peptide (ion-exchange chromatography, followed by HPLC, and then immunoaffinity chromatography), obtaining as a consequence high, pure lunasin-like fraction in comparison to the previously methods reported; however; this methodology may be complicated and the cost of production of the lunasin-like peptide may be raised if applied to an industrial level; moreover, the bioactivities of the peptide have been proven to be kept with a simpler purification process. Finally, further research is needed in order to obtain a clearer understanding of this barely peptide, as no sequence has been reported; it is proposed to determine the amino acid sequence of this peptide to confirm its similarity to that of soybean lunasin.

### 3.2. Lunasin-like Peptide in Wheat

Wheat is one of the most widely cultivated crops globally, valued not only for its nutritional content—including carbohydrates, proteins, lipids, and vitamins—but also for its bioactive compounds that may contribute to disease prevention and overall health [64].

Initial evidence for the presence of lunasin in wheat was reported by Jeong et al. [11], who identified the peptide using Western blotting and liquid chromatography–electrospray ionization mass spectrometry (LC-ESI-MS). They also observed that wheat-derived lunasin could be isolated at different stages of plant development and demonstrated its capacity to inhibit histone H3 and H4 acetylation in liver cells of rats fed with lunasin-enriched wheat. Similarly, Nakurte et al. [53] detected lunasin-like peptides in various wheat cultivars, with levels dependent on the developmental stage of the seeds.

However, these findings have since been questioned. Dinelli et al. [65], through in silico analyses of cereal transcript databases, reported an inability to identify sequences homologous to the soybean lunasin peptide in wheat and related cereals. Their investigation, which included LC-ESI-MS and PCR analysis across different wheat genotypes, failed to detect the presence of lunasin or lunasin-like peptides, casting doubt on earlier reports.

In light of this, Fan et al. [66] engineered wheat to express soybean-derived lunasin by cloning the relevant gene into the pCAMBIA3300 vector and introducing it into wheat via Agrobacterium-mediated transformation. Immunoassays confirmed the presence of lunasin in the transgenic plants, and the resulting peptides demonstrated a dose-dependent antiproliferative effect on HT-29 colon cancer cells.

Despite these advances, several limitations remain. The antibodies used to detect lunasin in wheat studies lacked comprehensive validation, and the mass spectrometry data only revealed partial peptide sequences that partially aligned with soybean lunasin. Complete sequence confirmation was not provided. Furthermore, Dinelli et al. [65] identified no homologous sequences in several wheat genotypes, underscoring the need for further validation of previously reported sequences. These discrepancies highlight the necessity of re-evaluating the protein sequences currently believed to contain lunasin-like peptides.

Ultimately, genetic modification may offer a viable approach for inducing lunasin expression in wheat, enabling better control over peptide production and concentration, as demonstrated in the transgenic models [13,66].

### 3.3. Lunasin-like Peptide in Rye

Rye is a cereal used in the food industry as a good source of starch, protein, and fiber. In addition, this cereal has several health benefits that have attracted the attention of several researchers [67]. In the literature, H.J. Jeong et al. [54] reported lunasin in rye identified by Western blot. In this study, rats were fed 1 µM rye enriched with lunasin, and inhibition of histone acetyltransferase (HAT) activities were observed in liver, kidney, and blood cells, as well as internalization of lunasin into the nucleus of mouse fibroblasts [54]. Moreover, in a study conducted by Nakurte et al. [53], lunasin was found in some varieties of rye, and its concentration was dependent on the stage of seed development (0.7–1.5 mg of lunasin-like peptide/g of grain) [53].

Rye has been reported to contain the lunasin-like peptide; nonetheless, the data on its identification could be complemented by adding the sequence of the protein and the use of different confirmation methods, because H.J. Jeong et al. [54] use HPLC and Western blot, but some specifications on the antibody used are missing, and Nakurte et al. [53] only used HPLC to compare the peaks of standard lunasin and the lunasin-like peptide in rye, which may raise concerns about the identification of the peptide to confirm if it is a similar sequence to that of lunasin or another peptide that exerts a similar activity.

### 3.4. Lunasin-like Peptide in Amaranth

Amaranth is part of the *Amaranthaceae* family, which shares characteristics of both a cereal and a legume seed, and it is considered a pseudocereal due to its amino acid composition [68,69,70].

It has been reported that amaranth seeds contain other substances that perform various biological functions in the diet with health benefits, which are called bioactive substances. These functions include protease inhibitors, antimicrobial peptides, lectins, antioxidant compounds, and compounds with anticancer properties [16]. It is important to highlight the glutelin and globulin 11S protein fractions for their content of SimLun, a peptide with 60% similarity to lunasin that has 43 amino acids, and which was found in the soybean 2S albumin storage protein [12] and whose cancer-preventive properties have been demonstrated [41].

Silva-Sánchez et al. [12] investigated the presence, characterization, and anticancer properties of the peptide lunasin in amaranth seeds. Enzyme-linked immunosorbent assay (ELISA) showed a mean concentration of 11.1 µg lunasin/g total protein extracted in four genotypes of mature amaranth seeds. The glutelin fraction had the highest lunasin concentration (3.0 µg/g), and trypsin-digested extracts showed induction of apoptosis against HeLa cells [12].

The anticancer activity of lunasin-like requires less time than soybean lunasin to internalize in the nucleus of NIH-3T3 cells and inhibits histone acetylation (H3 and H4 by 70 and 77%, respectively). Furthermore, it inhibited the transformation of NIH-3T3 cells into cancerous foci [9].

Montoya-Rodríguez et al. [59] compared the anti-inflammatory potential of unprocessed and extruded amaranth pepsin/pancreatin hydrolysates in lipopolysaccharide (LPS)-stimulated human (THP-1) and mouse (RAW 264.7) macrophages. The hydrolysates inhibited inflammation by preventing the activation of nuclear factor κB (NF-κB) signaling. Extrusion enhanced the anti-inflammatory effect of the hydrolysates in both cells by the production of bioactive peptides during processing [59].

Finally, Mazorra-Carrillo et al. [7] analyzed the changes in the proteomic profile of NIH-3T3 cells chemically transformed with the carcinogen 3-methylcholanthrene (3MC) in the absence or presence of the lunasin-like peptide, reducing it. In addition, the gel-based proteomic approach revealed new pathways of action and provided data on possible mechanisms of action of this bioactive peptide related to the NF-κB signaling pathway [7].

Amaranth is the seed with the most reports on the content of the lunasin-like peptide. Nevertheless, the studies of the activities reported for this peptide in amaranth [7,12,15,58] have been carried out with a protein hydrolysate and not the purified peptide due to the low concentration in natural resources; this may raise the question of whether this peptide or other(s) present the activity. In addition, the sequence was reported only for amaranth glutelins, and not for the rest of the protein fractions, but the evidence suggest that the lunasin-like peptide is present in all of them (albumins, globuins, prolamins, and glutelins); therefore, studies need to be conducted to sequence these fractions in order to compare it to the soybean lunasin peptide to confirm its presence. Furthermore, the use of a lunasin soybean antibody must be taken into account for future references, as it may not reflect the real concentration of this peptide due to a false positive reaction. Additionally, all the experiments related to the amaranth lunasin-like peptide were performed using hydrolysates, which can produce peptides with activities similar to those exerted by the lunasin peptide.

### 3.5. Lunasin-like Peptide in Triticale

Triticale is a cereal that has the characteristic of adapting to different environments and is used in human and animal feed. The crop has had greater development for animal feed, given the good grain quality and favorable dry matter yields compared to other fine-grained crops [71]. Nakurte et al. [53] reported the presence of a lunasin-like peptide in this cereal by LC-MS/MS assay, mentioning that triticale is the cereal with the best lunasin-like peptide content compared to wheat and barley species, which was also studied by them [53].

Another study by Galbas et al. [72] confirms the presence of a lunasin-like peptide in triticale by Western blot; furthermore, the antiproliferative activity of triticale lunasin-like peptide was analyzed in cervical cancer (HeLa) and ovarian cancer (SK-OV-3) cells, reducing their proliferation by 17% and 48%, respectively [72].

Nakurte et al. [53] and Galbas et al. [72] reported the presence of a lunasin-like peptide in triticale; however, the former only reported the content, and no activity was studied. This was conducted using HPLC coupled to mass spectroscopy, with no other methods used to confirm it. The latter performed a cytotoxic study with the purified fraction of the lunasin-like peptide and confirmation of its presence by Western blot, although not many specifications on the antibody used are given. Also, neither of the studies presented the sequence of this lunasin-like peptide to be compared to the soybean lunasin.

### 3.6. Lunasin-like Peptide in Oat

Oats, which belong to the *Poaceae* family, are an annual grass of Asian origin. Although oat cultivars are characterized by their relative protein concentration, little is known about genetic differences in elemental composition, which may also have nutritional importance for human and animal feed. Whole oats contain large amounts of valuable nutrients, such as soluble fiber, protein, unsaturated fatty acids, vitamins, minerals, and phytochemicals. The dietary fiber complex, with its antioxidants and other phytochemicals, is effective against cardiovascular diseases and some types of cancer [73].

Lunasin was first reported in oats by Nakurte et al. [52] by LC-MS/MS analysis. In this study, a comparison of chromatograms and mass spectra of lunasin obtained from five oat genotypes with the synthetic peptide lunasin was performed. In addition, the antioxidant activity of this oat lunasin was analyzed, which was similar to the activity presented by the synthetic lunasin [52].

In this article, the presence of lunasin was confirmed, and its antioxidant activity was tested and compared to the synthetic one. Nevertheless, no sequence of the peptide was performed, and no other reports on the presence of lunasin-like peptides in oats have been published.

### 3.7. Lunasin-like Peptide in Solanum

The genus Solanum (*Solanaceae* family) consists of more than 2000 species, which are distributed worldwide in tropical and subtropical regions. Numerous studies have reported the pharmacological activities of *S. nigrum* in recent decades. Various solvent extracts and bioactive compounds isolated from *S. nigrum* have shown many pharmacological properties [74]. In a study conducted by Jin et al. [56], lunasin-like peptide in crude extracts of five varieties of medicinal plants of Solanum origin and seven other important herbaceous plants were analyzed. Furthermore, the stability of digestion with pancreatin and pepsin was tested in vitro to measure the bioavailability of crude and autoclaved protein from Solanum. Inhibition of core histone acetylation was measured by a non-radioactive histone acetyltransferase (HAT) assay and a colorimetric HAT activity assay. In addition, the inhibitory effect of the Solanum lunasin-like peptide on retinoblastoma (Rb) protein phosphorylation was measured by immunoblotting against phospho-Rb. Sterilized lunasin-like peptide inhibited core acetylation of histones H3 and H4, HAT activities, and phosphorylation of Rb protein. Lunasin-like peptide in crude protein and sterilized crude protein was very stable for in vitro digestion with pepsin and pancreatin, whereas pure synthetic lunasin was digested within 2 min of reaction. Therefore, it is concluded that the lunasin-like peptide is a bioactive and bioavailable component in Solanum and its consumption may play an important role in cancer prevention [56].

In another study published by J.B. Jeong et al. [57], the protective effect of purified lunasin-like peptide from *Solanum nigrum* L. on DNA against oxidative stress was reported. The lunasin-like peptide protected DNA from oxidative damage. The protective effect of this lunasin-like peptide is due to its ability to chelate Fe^2+^, scavenge the generated hydroxyl radical, and block the generation of hydroxyl radical by the same chelation of Fe^2+^ ion. Therefore, Solanum lunasin-like peptide may play an important role in the chemoprevention of oxidative carcinogenesis [57]. However, the sequence of the lunasin-like peptide from Solanum may be needed in order to make a better comparison to that of the soybean lunasin.

### 3.8. Lunasin-like Peptide in Legumes

Legumes are dried seeds of plants of the Fabaceae family, of which a large number of species and varieties are used as food for both humans and animals [75]. Nutritionally, legumes contain a significant proportion of protein and are attributed with health benefits such as therapeutic and protective effects for chronic diseases such as cancer, diabetes, and obesity [76]. However, there is little data on their chemical and technological characteristics and their fermentation. The latter has been recognized as the most effective tool to improve the nutritional and functional properties of flours. In a study conducted by Rizzello et al. [55], the presence of lunasin or similar peptides in various traditional Italian legumes (*Phaseolus vulgaris*, *Cicer arietinum*, *Lathyrus sativus*, *Lens culinaris*, and *Pisum sativum* species), whose flours were fermented or not with lactic acid bacteria (*Lactobacillus plantarum* C48 and *Lactobacillus brevis* AM7), was investigated. An integrated approach based on chemical, immunological, and ex vivo (human adenocarcinoma Caco-2 cell cultures) analyses were used to demonstrate the physiological potential of lunasin-like polypeptides [55].

The extracts of the unfermented legume doughs and the mother doughs were subjected to Western blot analysis, using an anti-lunasin primary antibody, finding an absence of this, but finding different immunoreactive peptides (nine similar to lunasin); in addition, the concentration of immunoreactive peptides increased by the proteolysis caused during fermentation. The extracts of the plants showed inhibition on the proliferation of Caco-2 cells, especially those of Fagiolo di Lamon, Cece dell’Alta Valle di Misa, and Pisello riccio di Sannicola, which showed a decrease in cell viability of up to 70% [55]. In this case, the fermentation process produced several peptides. Some are hypothesized to be lunasin-like peptides, but no real correlation to this is presented, just the reaction of the lunasin antibody with many of these to identify it; however, this should be further studied to confirm if the activity is exerted by this lunasin-like peptide or by other peptides produced during the fermentation.

### 3.9. Lunasin-like Peptide in Maize

Maize is among the three most important cereals worldwide, with key applications in human and animal nutrition, as well as in the production of biofuels. It is also a key raw material in the processed food and beverage industries [77]. This crop is a staple food for more than one billion people, in whose diet the grain can provide more than 50% of total caloric energy [78].

In 2024, Hao et al. [13] produced a transgenic maize that overexpressed lunasin; this was conducted by using the YF464 maize variety as a receptor, obtaining 13 overexpression lines. The maize lunasin was identified and purified by Western blot and ultrahigh-pressure liquid chromatography–electrospray ionization with tandem mass spectrometry (UPLC-ESI-MS/MS). Moreover, maize lunasin presented higher antioxidant, anti-inflammatory, and anticancer activities and better antiproliferation activity on the breast cancer cell line MDA-MB-231 [13]. Here, the lunasin was not naturally present in maize; the genetic modification was performed to overexpress lunasin with a similar structure and characteristics to those presented in soybean lunasin, thus giving the same activities as this one but raising the concentration of lunasin produced in this crop. This methodology can be used to provide a peptide with the same sequence, characteristics, and bioactivities that allow better experiments with reproducibility, and therefore, to obtain better knowledge of the pathways that are related to this peptide synthesis, but with the advantage of increasing the lunasin expression and concentration.

## 4. Controversy on Lunasin-like Peptide in Different Plant Sources

There is some controversy about whether the peptides reported on seeds other than soybeans are similar to the lunasin peptide contained in the latter. Alaswad and Krishnan [79] presented a study where an immunological investigation was carried out on various seeds, using polyclonal antibodies against the N-terminal region of 20 amino acids, with sequence SKWQHQQQQDSCRKQLQGVNLT, and against the C-terminal region of 15 amino acids, with sequence CEKHIMEKIQGRGDD, both regions for soybean lunasin. The results revealed that several proteins of the wheat, rye, barley, triticale, coriander, sesame, anise, black cumin, okra, sunflower, castor bean, and flax seeds react against the antibodies against the N-terminal region of lunasin, but do not react against the antibodies of the C-terminal region for most of the seeds studied; some did not react with any of the antibodies; this is when the immunological study was carried out in extracts of albumins and total proteins of the seeds. In addition, a Western blot was performed on these fractions, and the reactivity with the antibodies was tested again, obtaining a reaction against the antibodies for the N-terminal region with a few proteins of diverse seeds, but without reactivity against the C-terminal antibody. The null reactivity with the antibody for the C-terminal region of lunasin proves that this peptide is either not present in the seeds or is found in concentrations below the assay’s detection limit [79]. Structure–function analyses indicate that lunasin’s bioactivity relies on distinct yet complementary peptide regions. The C-terminal poly-aspartic tail is indispensable for chromatin-binding and epigenetic regulation, as demonstrated by studies showing its ionic interaction with deacetylated histones H3 and H4; deletion or scrambling of this acidic domain markedly reduces histone association and chemopreventive effects. The central RGD motif facilitates internalization and integrin engagement, and its mutation (e.g., RGD→RAD) abolishes uptake-dependent activities such as inhibition of oncosphere formation. Meanwhile, the N-terminal and central helical domain provides structural contributions to histone affinity and specificity, complementing the poly-D tail in stabilizing interactions with chromatin. Consistently, alterations in any of these modules diminish activity, and although some truncated fragments (e.g., the poly-D tail alone) retain partial effects, full-spectrum functionality generally requires the intact peptide [19,20,32]. Therefore, the complete structure gives lunasin its bioactive properties.

Another point to consider is the type of antibodies used for the various immunoidentification studies. The data on these antibodies is not provided; it is only mentioned in some articles that they are rat monoclonal or polyclonal antibodies against soybean lunasin. However, it is not mentioned whether they were developed against some regions of lunasin or the entire sequence, and the specificity of these lunasin antibodies is not mentioned either [79], being able to react with other proteins with a similar primary sequence or a conformational sequence in a three-dimensional space. In the case of the immuno-study mentioned above, the antibody to the N-terminal region of lunasin reacted nonspecifically against other proteins under non-restrictive conditions. Although the nonspecific binding can be reduced or eliminated by increasing the concentration of Tween-20 during Western blotting [79] this was conducted in the study with a concentration of 0.5% Tween-20, reducing the nonspecific binding with proteins quite a lot. However, there is an article [56] where they use a 1% concentration of Tween-20, where high reactivity with antibodies for the N-terminal region of lunasin was obtained, which is attributed to differences in the quality of the antibodies used for the studies [79]. Moreover, specific antibodies for each lunasin-like peptide in each type of seed must be produced in order to identify them and to avoid false positives; in addition, other studies of structure and sequence can be carried out along with this to confirm the presence of these lunasin-like peptides.

There is another previously mentioned study where Dinelli et al. [65] analyzed several sequences of wheat proteins in databases. This study found that none of the sequences reported for wheat had a similarity with the sequence for the lunasin peptide. Detailed searches of transcriptome databases and cereal DNA sequences failed to identify lunasin-coding sequences, raising doubts as to whether these peptides are lunasin-like or not. To corroborate the presence of lunasin in various plant species, matrix-assisted laser desorption ionization (MALDI) and LC-ESI-MS were used to obtain the peptide sequence of lunasin-like peptides in barley and wheat [11,54,63], which matched the lunasin sequences of soybean. This observation prompted the investigation of transcriptome and DNA sequence databases of plant species in which this peptide is reported, in which no sequences encoding the lunasin peptide were found to be present [80]. This presents another challenge to overcome since several articles reporting the presence of peptides similar to soybean lunasin do not present sequencing studies of these peptides, which are required to confirm that they are similar to lunasin, and no other peptide or peptides are exerting these activities.

Finally, the study by Rizzello et al. [55] investigated the presence of lunasin in Italian legumes by Western blot analysis, without finding the presence of lunasin in them. This same study presented another possibility of how positive reactions to high molecular weight proteins can be obtained with lunasin antibodies, since from these high molecular weight proteins, lunasin-like peptides can be released by hydrolysis, in this case, during fermentation of doughs made with the flours of these legumes [55]. This can be transpolated to other sources of plant origin, where hydrolysis of high molecular weight proteins results in the release of soybean lunasin-like peptides, presenting a higher reactivity against lunasin antibodies.

Furthermore, this raises the question of whether or not the peptides liberated during germination, fermentation, or hydrolysis may be the ones that present antioxidant, anticarcinogenic, antimutagenic, or immunomodulatory activities and not a lunasin-like peptide and these same peptides may react due to random interactions that may form the sequence recognized by the anti-lunasin antibodies, which may result in a false positive as it was in the case in wheat [11,65]. This has been previously described in different studies for cereals and legumes, in which these activities have been studied [81,82,83,84,85,86,87,88,89,90,91,92,93,94,95,96,97]. Peptides rich in hydrophobic amino acids (Tyr, Try, Phe, Leu, Ile, and Ala) have been described to act as hydrogen donors, blocking the peroxidation chain reaction caused by free radicals [98]. Also, peptides that contain Asp, Glu, Ser, Pro, Ala, Ile, Leu, Phe, and Tyr exert antioxidant activity due to their ability to chelate ferrous ions and scavenge other free ions [99]. For immunomodulatory activity, peptides containing Gln are used as a raw material to synthesize glutathione to promote lymphocyte proliferation and improve macrophage phagocytosis, thus regulating immune function [93]. Regarding the anticancer properties, many pathways have been studied where peptides exert this activity; however, histones and histone deacetylases are very important targets due to the induction of cell cycle arrest, autophagy, necrosis, and apoptosis [100]. This happens in two different ways: the first is by the inhibition of histone deacetylase (HDAC), which is usually achieved by cyclic peptides that occur naturally, and other compounds [101], and the other is by interacting with the histones, where amino acids with negative charge do not allow a closed chromatin formation, and therefore, induce cell cycle arrest and apoptosis [102]. Peptides containing the previously mentioned amino acids are produced during the processes of fermentation, germination, and hydrolysis; therefore, this has to be taken into account in the study of peptides similar to lunasin to try to define whether the lunasin-like peptide is the one producing the activity or if it is another peptide (Table 1).

## 5. Future Perspective

Numerous advances have been made in the search for peptides with important bioactivities, particularly in the identification of peptides with anticancer and antioxidant activities. Soy lunasin-like peptides have become of interest in research because they exhibit these activities, so their identification and testing to prove their effectiveness is of utmost importance. At present, several articles mention the identification of this type of peptide using antibodies designed to recognize the lunasin peptide; however, it is advisable to use an antibody that specifically works for the sequence of the lunasin-like peptide that is being studied. In addition, it is necessary to obtain the sequence of the proteins where these peptides are found in order to develop an appropriate method that allows their identification in the different seeds/grains. This, in turn, will allow comparison with the lunasin peptide to corroborate their presence and percentage of similarity, eliminating the possibility that these activities are present in other peptides or are due to conformational peptides.

Moreover, there are some papers where the purification of the lunasin peptide was not carried out, and a hydrolysate was used to study cytotoxic effects on cancer cell lines [7,12,15], which may bring into consideration studying and finding or confirming which peptide or peptides are the ones that exert this anticancer activity, because the hydrolysate not only contains the sequence of lunasin-like peptides, but also other peptides that can possess this activity. Furthermore, the lunasin-like peptide may be hydrolyzed by this enzymatic reaction, producing other peptides that may or may not have anticancer activities.

Another approach that can be carried out is the production of a lunasin or a lunasin-like peptide using genetic modification of the microorganisms used in precision fermentation. This would bring the advantages of having the same sequence every time the peptide is produced, reducing in that way the variability presented in the studies that have been conducted. In addition, this would allow us to perform other studies with better reproducibility and repeatability, overcoming the discrepancies presented in previous studies. Additionally, recombinant production of lunasin offers a promising industrial route: optimized lunasin genes expressed in *Escherichia coli* as fusion proteins (e.g., with cellulose-binding domains or His-tags) achieve soluble expression, protease cleavage yields the authentic peptide, and purification provides material in the hundreds of milligrams per liter range [103,104]. Recombinant systems thus combine scalability, lower costs, and have the possibility of engineering analogs, positioning them as the most viable industrial strategy compared to extraction and synthesis routes.

It is also important to try to define the pathways by which these different peptides act and how they manage to exhibit their bioactivities. There is little information regarding the pathways of action of these peptides, and in most cases, it is assumed that it is due to the same pathways studied for the lunasin peptide; however, this information should be verified in order to obtain better knowledge and understanding of the mechanism of action of these molecules that will allow us to make optimal use of their properties.

Finally, there is a special need to decide to what extent a peptide can be called lunasin-like, as there is no reference on this, and peptides with less than 50% similarity to lunasin have been called lunasin-like peptides.

## 6. Conclusions

Lunasin-like peptides have been identified in a variety of plant sources, including barley, wheat, rye, triticale, oats, black belladonna, amaranth, beans, chickpea, grass pea, lentils, and pea. These peptides have been associated with anticancer and antioxidant properties in several cases. However, it is likely that, following ingestion, they undergo enzymatic hydrolysis, leading to the formation of conformationally active fragments that may account for the reported bioactivities. Although numerous studies report the presence of lunasin-like peptides in seeds, few have provided definitive structural identification. The biological activities observed may therefore be attributed to other peptides with antitumor or antiproliferative effects unrelated to lunasin. To clarify this, a detailed characterization of the peptides involved is necessary. Consequently, peptide sequencing is essential to confirm their structural similarity to soy-derived lunasin. Moreover, current studies do not adequately address the gastrointestinal stability, accessibility, or bioavailability of these peptides post-ingestion, highlighting a critical gap in understanding their physiological relevance.

## Data Availability

No new data were created or analyzed in this study. Data sharing is not applicable to this article.
